# Adherence to support pessary in the treatment of pelvic organ prolapse: a retrospective study conducted among 1,371 women

**DOI:** 10.1007/s00192-023-05616-z

**Published:** 2023-08-07

**Authors:** Cecilie Helstrup Brandt, Mahsa Yamolaei, Chunsen Wu, Ulla D. Hansen, Vibeke Rasch

**Affiliations:** 1 Medical Faculty, University of Southern Denmark, 55 Campusvej, 5230 Odense, Denmark; 2 Department of Gynecology and Obstetrics, Odense University Hospital, Odense, Denmark; 3 Department of Clinical Research, University of Southern Denmark, Odense, Denmark

**Keywords:** Pelvic organ prolapse, Pessary, Discontinuation

## Abstract

**Introduction and hypothesis:**

The objective was to investigate the adherence to pessary treatment in women with pelvic organ prolapse (POP) who were found eligible for this treatment by the urogynecologist, at the first visit at the Department of Gynecology and Obstetrics, Odense University Hospital.

**Methods:**

Data were extracted from the women’s medical records. Frequency tabulations were performed to describe the women’s reasons for pessary discontinuation by age group. Binominal logistic regression analysis was conducted to investigate how women’s age, POP characteristics, urogynecological history, and their pessary experience and management were associated with continued pessary use.

**Results:**

This study included 1,371 women treated with support pessary. Of these, 850 women continued pessary treatment and 521 women underwent surgical treatment. A history of hysterectomy (OR: 0.68, 95% CI: 0.51–0.90, *p* = 0.008), urinary incontinence (OR: 0.71, 95% CI: 0.56–0.89, *p* = 0.003), and previous pessary use (OR: 0.75, 95% CI: 0.56–0.99, *p* = 0.047) were significant factors associated with discontinuation. Further, women aged 81–99 years were significantly more likely to continue pessary treatment (OR: 1.77, 95% CI: 1.15–2.74, *p* = 0.009). “POP surgery,” “prolapse stage,” and “prolapse predominant compartment” were not associated with discontinuation. Approximately 38% of women aged 26–54 years discontinued owing to personal preference.

**Conclusions:**

Hysterectomy, incontinence, and previous pessary use are significant predictors of pessary discontinuation. Increasing age is significantly associated with pessary continuation.

## Introduction

Pelvic organ prolapse (POP) is a relatively common medical condition, and the prevalence increases with age [1]. Studies indicate that approximately 30–40% of women suffer from POP to some extent [2, 3]. Symptoms include vaginal, defecatory, urinary, and sexual symptoms [3–6]. Indication for treatment is based on the severity of symptoms and is individualized depending on prolapse stage and patient preference [5, 7]. The choice of treatment can be surgical or nonsurgical [3, 6]. Nonsurgical treatments involve pessaries, pelvic floor muscle exercise and vaginal estrogen treatment [1, 6, 8]. Compared with surgery, a vaginal pessary is a cheap and minimally invasive treatment [4, 6], and can be inserted by a health care professional and maintained by the patient [5, 9, 10]. Although pessaries are effective in reducing POP-related symptoms [9, 11], some women are not candidates for pessary use owing to personal preferences and complications such as erosions, discomfort, vaginal discharge, and improper pessary fitting [5, 7]. Although surgery has a high success rate in minimizing POP, it is associated with a re-operation rate of up to 30% [12]. According to the clinical guidelines from the Danish Urogynecological Association, women should be offered pessary treatment before proceeding to surgery [13]. The aim of this single-center retrospective descriptive study is to investigate the reasons for discontinuation of pessary use and assess factors associated with adherence to pessary treatment in women with POP.

## Materials and methods

### Study design and participants

**Fig. 1:**
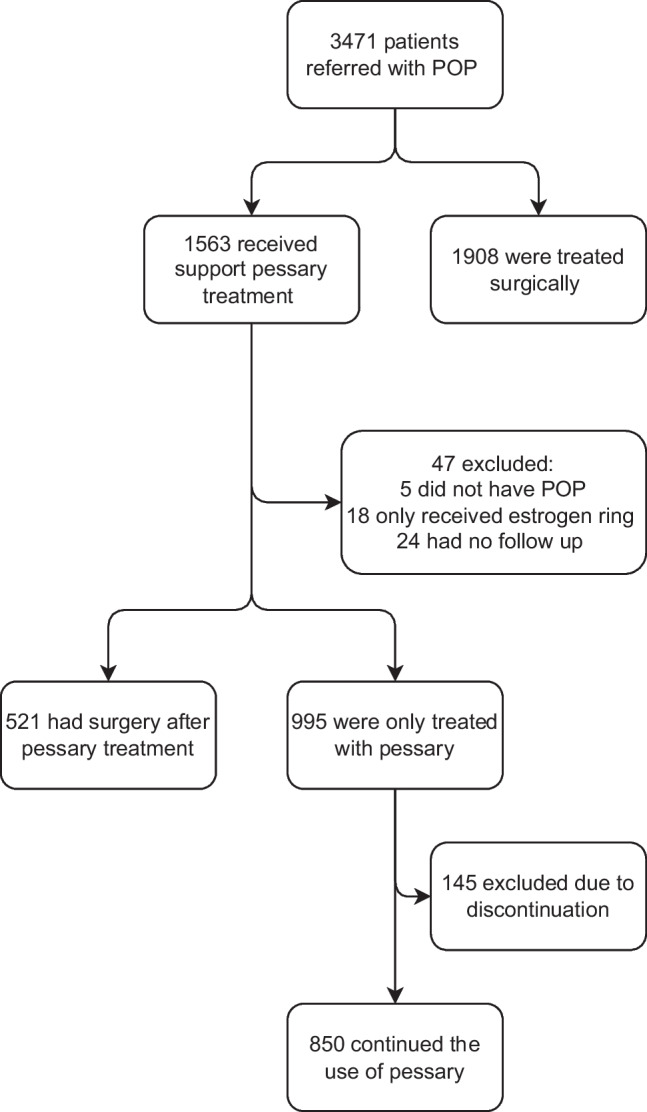
Flowchart illustrates the included and excluded women

A retrospective descriptive study conducted in Denmark including women with POP treated with support pessary. Data were collected at the Department of Gynecology and Obstetrics, Odense University Hospital (OUH). This study included women who, according to the International Classification of Disease (ICD10), were registered with the diagnosis code for POP (DN81* and DN993), and the procedure codes for pessary treatment (BJDZ* and BJDA*) and surgical treatment (KLEF* and KLCD) respectively. A total of 3,470 women had the POP diagnosis code within a 5-year timeframe, of which 1,908 women received surgery as first-line treatment based on the urogynecologist’s assessment of the women’s eligibility or the patient declining pessary treatment. Thus, 1,563 women underwent pessary treatment, 192 of these women were excluded from the analysis, either because of a lack of POP description (*n* = 5), only estriol vaginal ring treatment (*n* = 18), no follow-up consultation after pessary placement (*n* = 24), or incomplete data in the patient medical records (*n* = 145). Data on pelvic floor physiotherapy is not included in the study as it is not routinely offered at OUH as a first-line treatment for POP. This resulted in a population of 1,371 women, of whom 850 continued pessary treatment and 521 opted for surgical treatment. The included and excluded women are all depicted in Fig. 1.

### Data registration and variables

Data were collected from the women’s electronical medical record, which was obtained using their unique personal identification number. Data were registered manually in Microsoft Excel and included information on “age,” “prolapse stage in the anterior,” “apical,” and “posterior compartment,” “incontinence,” “hysterectomy,” “previous prolapse surgery,” “previous use of pessary,” “previous incontinence surgery,” “sexual activity,” and “self-administration of pessary.” It was assumed that the women had continued pessary treatment if they had claimed to be satisfied at their last follow-up consultation. Furthermore, the categories: “incontinence,” “self-administration,” and previous surgery were marked as “no” during data registration if not stated otherwise. No assumptions were made concerning sexual activity and it was therefore registered as unknown if it was not noted in the medical records. The reasons for pessary discontinuation were categorized as: “erosions,” “discomfort/pain,” “discharge issues,” “personal preference,” “insufficient effect” (meaning that the pessary could not sufficiently keep the prolapse in place or the prolapse symptoms were not reduced), “pessary dislodgement,” and “incontinence/defecatory problems.” The date on which the women gave their consent to surgery was registered as the end date for pessary treatment.

### Statistical analysis

Statistical analysis was performed using StataBE version 17. Baseline characteristics were presented as total number (%) for categorical variables. To investigate the impact of different variables on pessary adherence the population was categorized into two groups: continuation (*n* = 850) and discontinuation (*n* = 521). Binominal logistic regression analysis was conducted to estimate odds ratio (OR) with 95% confidence interval and equivalent *p* value to determine the association between the baseline characteristics and pessary discontinuation. “Continued with pessary” and “discontinued pessary for surgery” were defined as the dependent dichotomous variables. “POP compartment,” “POP stage,” “age,” “previous surgery,” “incontinence,” “sexual activity,” “self-management,” and “previous pessary” were defined as the independent variables. Crude ORs were calculated followed by age-adjusted analyses. The *p* value was considered statistically significant if < 0.05.

### Ethics

Permission to obtain data through the medical journals was granted by the Region of Southern Denmark. Data were stored in accordance with the European Union regulations for storage of personally sensitive data.

## Results

This study included a population of 1,371 POP women treated with support pessary within a 5-year period. Among these, a total of 850 (56.1%) continued pessary treatment. The mean age for the women in the pessary group was 70.9 years, whereas it was 68.5 years in the surgical group. Women who underwent surgery would use the pessary for approximately 11.5 months on average. Only 14.9% in the pessary group and 19.8% in the surgery group reported being sexually active (Table 1).

**Table 1:** Baseline characteristics

	Total (*N* = 1,371)	Anterior (iso) *n* 469 (34.2%)	Apical (iso) *n* = 83 (6%)	Posterior (iso) *n* = 34 (2.5%)	Combined *n* = 785 (57.3%)
Age, years, *n* (%)					
26–54	145 (10.6)	43 (9.2)	17 (20.5)	6 (17.7)	79 (10.1)
55–64	222 (16.2)	77 (16.4)	12 (14.5)	4 (11.8)	129 (16.4)
65–70	254 (18.5)	84 (17.9)	16 (19.3)	3 (8.8)	151 (19.2)
71–75	282 (20.6)	90 (19.2)	17 (20.5)	7 (20.6)	168 (21.4)
76–80	244 (17.8)	86 (18.3)	10 (12)	3 (8.8)	145 (18.5)
81–99	224 (16.3)	89 (19)	11 (13.3)	11 (32.3)	113 (14.4)
POP stage, *n* (%)					
1	31 (2.3)	15 (3.2)	1 (1.2)	6 (17.7)	9 (1.1)
2	712 (51.9)	309 (65.9)	36 (43.4)	19 (55.9)	348 (44.3)
3	481 (35.1)	129 (27.5)	29 (34.9)	8 (23.5)	315 (40.1)
4	126 (9.2)	8 (1.7)	17 (20.5)	1 (2.9)	100 (12.7)
Missing data	21 (1.5)	8 (1.7)	0	0	13 (1.7)
Hysterectomy, *n* (%)					
Yes	229 (16.7)	84 (17.9)	12 (14.5)	12 (35.3)	121 (15.4)
No	1.142 (83.3)	385 (82.1)	71 (85.5)	22 (64.7)	664 (84.6)
Previous POP surgery, *n* (%)					
Yes	173 (12.6)	76 (16.2)	7 (8.4)	10 (29.4)	80 (10.2)
No	1.198 (87.4)	393 (83.8)	76 (91.6)	24 (70.6)	705 (89.8)
Incontinence surgery, *n* (%)					
Yes	14 (1)	8 (1.7)	1 (1.2)	0	5 (0.6)
No	1.357 (99)	461 (93.3)	82 (98.8)	34 (100)	780 (99.4)
Incontinence, *n* (%)					
Yes	497 (36.3)	180 (38.4)	25 (30.1)	10 (29.4)	282 (35.9)
No	874 (63.7)	289 (61.6)	58 (69.9)	24 (70.6)	503 (64.1)
Previous pessary, *n* (%)					
Yes	253 (18.5)	100 (21.3)	15 (18.1)	6 (17.7)	132 (16.8)
No	1.118 (81.5)	369 (78.7)	68 (81.9)	28 (82.3)	653 (83.2)
Self-administration, *n* (%)					
Yes	276 (20.1)	96 (20.5)	23 (27.7)	10 (29.4)	147 (18.7)
No	1.095 (79.9)	373 (79.5)	60 (72.3)	24 (70.6)	638 (81.3)
Sexual activity, *n* (%)					
Yes	230 (16.8)	56 (11.9)	18 (21.7)	10 (29.4)	118 (15)
No	187 (13.6)	58 (12.4)	9 (10.8)	2 (5.9)	146 (18.6)
Missing data	954 (69.6)	355 (75.7)	56 (67.5)	22 (64.7)	521 (66.4)

The three most common reasons for discontinuation of pessary use were insufficient effect (19%), ring dislodgement (19.9%), and personal preference (19%). Discomfort/pain and excessive discharge constituted the most common combination of symptoms that led to discontinuation. Amongst those aged 26–54 and 55–64 years the reason for discontinuation was mainly personal preference (37.7% and 34% respectively). In comparison, 10.3%, 18.7%, 12.1%, and 5.2% in the remaining four age groups reported this reason for discontinuation. On average women aged 65 and above primarily discontinued owing to insufficient effect (22%) and ring dislodgement (23.8%). More details on reasons for discontinuation are shown in Table 2.

**Table 2:** Distribution of reasons for pessary discontinuation

Age in years	Discomfort/pain, *n* (%)	Erosions, *n* (%)	Discharge, *n* (%)	Personal preference, *n* (%)	Insufficient effect, *n* (%)	Dislodgement, *n* (%)	Incontinence/ defecatory problems, *n* (%)	Total, *N* (%)
26–54	17 (24.6)	1 (1.4)	4 (5.8)	**26 (37.7)**	13 (18.8)	6 (8.7)	2 (2.9)	69 (100)
55–64	21 (19.8)	5 (4.7)	7 (6.6)	**36 (34)**	9 (8.5)	14 (13.2)	14 (13.2)	106 (100)
65–70	16 (13.7)	11 (9.4)	12 (10.3)	12 (10.3)	**30 (25.6)**	27 (23.1)	9 (7.7)	117 (100)
71–75	15 (14)	12 (11.2)	5 (4.7)	20 (18.7)	17 (15.9)	**26 (24.3)**	12 (11.2)	107 (100)
76–80	14 (13.1)	13 (12.1)	13 (12.1)	13 (12.1)	**22 (20.6)**	**22 (20.6)**	10 (9.3)	107 (100)
81–99	7 (9.1)	13 (16.9)	6 (7.8)	4 (5.2)	20 (26)	**21 (27.3)**	6 (7.8)	77 (100)
In total	90 (15.4)	55 (9.4)	47 (8.1)	111 (19)	111 (19)	116 (19.9)	53 (9.1)	583^a^ (100)

The entries in bold text represent the most common reason for pessary discontinuation for each age group

^a^There are two reasons noted for 62 of the 521 women, in all 583

The binominal logistic regression analysis indicated that women aged 81–99 were significantly more likely to favor pessary treatment (OR: 1.77, 95% CI: 1.15–2.74, *p* = 0.009). The likelihood of favoring pessary treatment significantly decreased if the women had had a hysterectomy (OR: 0.68, 95% CI: 0.51–0.90, *p* = 0.008) or had had incontinence (OR: 0.71, 95% CI: 0.56–0.89, *p* = 0.003). In the age-adjusted analysis, hysterectomy (OR: 0.64, 95% CI: 0.47–0.85, *p* = 0.002), incontinence (OR: 0.71, 95% CI: 0.56–0.89, *p* = 0.003), and previous pessary use (OR: 0.75, 95% CI: 0.56–0.99, *p* = 0.047) were significantly associated with pessary discontinuation. The statistics are depicted in Table 3.

**Table 3:** Binominal logistic regression of continued and discontinued pessary treatment

	Pessary continuation, *N* (%) = 850	Pessary discontinuation, *n* = 521	OR (95% CI)	*p* value	aOR (95% CI), adjusted for age	*p* value
Compartment, *n* (%)						
Anterior	294 (62.7)	175 (37.3)	1		1	
Apical	49 (59)	34 (41)	0.86 (0.53–1.38)	0.528	0.89 (0.55–1.45)	0.659
Posterior	21 (61.8)	13 (38.2)	0.96 (0.47–1.97)	0.915	0.92 (0.44–1.90)	0.836
Combined	486 (61.9)	299 (38.1)	0.97 (0.76–1.23)	0.784	0.98 (0.77–1.45)	0.881
1 compartment	364 (62.1)	222 (37.9)	1		1	
≥ 2 compartments	486 (61.9)	299 (38.1)	0.99 (0.80–1.24)	0.938	1.00 (0.8–1.25)	0.987
POP stage, *n* (%)						
1	22 (71)	9 (29)	1		1	
2	432 (60.7)	280 (39.3)	0.63 (0.87–1.39)	0.264	0.62 (0.28–1.38)	0.241
3	297 (61.7)	184 (38.3)	0.66 (0.3–1.47)	0.307	0.61 (0.27–1.35)	0.221
4	78 (61.9)	48 (38.1)	0.66 (0.28–1.56)	0.349	0.57 (0.24–1.36)	0.204
Missing data	21 (100)	0				
Age (years), *n* (%)						
26–54	81 (55.9)	64 (44.1)	1			
55–64	125 (56.3)	97 (43.7)	1.02 (0.67–1.55)	0.933		
65–70	150 (59.1)	104 (40.9)	1.14 (0.75–1.71)	0.534		
71–75	183 (64.9)	99 (35.1)	1.46 (0.97–2.2)	0.069		
76–80	156 (63.9)	88 (36.1)	1.40 (0.92–2.13)	0.115		
81–99	155 (69.2)	69 (30.8)	1.77(1.15–2.74)	0.009*		
Previous POP surgery, *n* (%)						
Yes	104 (60.1)	69 (39.9)	0.91 (0.66–1.27)	0.585	0.90 (0.65–1.25)	0.541
No	746 (62.3)	452 (37.7)	1		1	
Hysterectomy, *n* (%)						
Yes	124 (54.2)	105 (45.9)	0.68(0.51–0.90)	0.008*	0.64 (0.47–0.85)	0.002*
No	726 (63.6)	416 (36.4)	1			
Incontinence surgery, *n* (%)						
Yes	9 (64.3)	5 (35.7)	1.1 (0,37–3,31)	0.859	1.11 (0.37–3.35)	0.855
No	841 (62)	516 (38)	1		1	
Incontinence, *n* (%)						
Yes	282 (56.7)	215 (43.3)	0,71(0.56–0.89)	0.003*	0.71 (0.56–0.89)	0.003*
No	568 (65)	306 (35)	1		1	
Previous pessary, *n* (%)						
Yes	149 (58.9)	104 (41.1)	0.85 (0.65–1.13)	0.260	0.75 (0.56–0.99)	0.047*
No	701 (62.7)	417 (37.3)	1			
Self-administration, *n* (%)						
Yes	176 (63.8)	100 (36.2)	1.1 (0.86–1.45)	0.498	1.29 (0.96–1.73)	0.092
No	674 (61.6)	421 (38.5)	1			
Sexual activity, *n* (%)						
Yes	127 (55.2)	103 (44.8)	0.88 (0.60–1.30)	0.529		
No	109 (58.3)	78 (41.7)	1			
Missing data	614 (64.4)	340 (35.6)				

The table illustrates crude and age-adjusted odds ratio (*OR*) with equivalent 95% confidence interval (*95% CI*) and *p* value for the baseline characteristics

1 indicates reference rate

*Indicates statistical significance (*p* < 0.05)

## Discussion

A key finding in the study was that continuation of pessary treatment was significantly lower in younger women and in women who were hysterectomized, had preexisting incontinence, and women who had previously been treated with pessary. The study suggests that the three main complications leading to pessary discontinuation were ring dislodgement, insufficient effect, and personal preference. The latter was mainly dominated by the youngest age groups.

In line with other studies, women who are hysterectomized are significantly more likely to undergo surgery [14, 15]. The same significant tendency is stated by Mutone et al. [14] and Patnam et al. [15] who describe that women with a history of hysterectomy are more likely to discontinue pessary treatment. Contrastingly, Ma et al. [16] concluded that pessaries remain useful in hysterectomized women. This indicates the importance of finding the right size and type of pessary for women who have had a hysterectomy as they might also have a chance of effective pessary treatment. No significant association between previous POP surgery and discontinuation of pessary treatment was found, although other studies suggest that previous POP surgery is significantly associated with discontinuation [8, 9, 14, 16–18]. This association might not show owing to the urogynecologist’s selection of eligible patients for pessary treatment. The association between previous POP surgery and discontinuation might also be caused, like hysterectomy, by the anatomical changes that complicate pessary treatment.

The study finds that women with incontinence, upon beginning pessary treatment, are significantly more likely to have surgery. Furthermore, it was discovered that women with previous pessary use have a significant tendency to undergo surgery. Although these associations are significant, this study does not include sufficient data to explain this occurrence. Thus, further studies are needed to elaborate the cause.

No significant association between discontinuation and prolapse stage was found in this study, whereas other studies do highlight that advanced prolapse stage is associated with discontinuation of pessary treatment [8, 9, 19, 20]. Additionally, no correlation between pessary discontinuation and predominant prolapse compartment was found. In comparison, Ma et al. and Manzini et al. [9, 21] stated that a prolapse in the posterior compartment is a risk factor for unsuccessful fitting and Coelho et al. and Cheung et al. [18, 20] found that an apical compartment prolapse increases the risk of unsuccessful pessary fitting.

This study found that the three main rationales for pessary discontinuation were “pessary dislodgement,” “insufficient effect,” and “personal preference.” This was comparable with Albuquerque Coelho et al. [11] who found that “personal preference,” “pessary dislodgement,” and “discomfort” were the main reasons for discontinuation. In our study, approximately 38% of women in the youngest age group discontinued pessary treatment owing to personal preference. Other studies found that younger women were significantly more likely to ultimately choose surgical treatment [8, 19, 22]. Younger women might opt for surgical management owing to the ongoing treatment and the fact that they must either self-manage or visit the clinic. Another possible explanation was that pessary treatment complicates sexual activity. Vasconcelos et al. [23] found that a strong predictor for the discontinuation of pessary treatment was sexual activity. However, owing to incomplete data, no conclusion could be drawn regarding the influence of sexual activity on pessary treatment. In contrast, the reason for discontinuation in the older age groups tended to be either “pessary dislodgement” or “insufficient effect.” Furthermore, our study found a nonsignificant tendency that women 71 years and above were likely to adhere to pessary treatment. Similarly, Umachanger et al. and Dengler et al. [8, 24] both concluded that increasing age was associated with pessary continuation, thus supporting our finding. This indicated that older women were likely to continue pessary treatment provided that the pessary was effective in relieving the symptoms and that the fitting was appropriate.

Although many studies have shown promising results regarding pessary treatment [4, 6, 11, 16, 17, 20, 22], this study illustrated that 40% of women treated with pessary as first-line treatment discontinued. This indicates that finding significant predictors for successful pessary treatment can help the clinician when recommending the ideal treatment. In contrast to other studies, this study identified incontinence as a significant predictor of pessary discontinuation. There is no clear explanation of this observation and thus more research should be made to improve the knowledge regarding the association among POP, incontinence, and pessary adherence.

A limitation of this study is that the population was found eligible for, and accepted, pessary treatment, which can lead to differences when the results are compared with studies that have an unselected study population. Data were collected retrospectively, causing subjective interpretation of the patient journals and missing data. Major variations in both the structure and the content of the medical journals forced us to make assumptions. In addition, doctors with varying levels of experience performed the vaginal examinations, which may bias the results owing to interdoctor reliability. Furthermore, all data were registered manually, which increased the risk of typing errors. Another limitation of this study is the lack of access to patient data from private practitioners and clinics, as important information regarding complications, continuation, and discontinuation may be missing. This leads to our assumption that women continue pessary treatment if they reported that they were satisfied with their treatment during the last follow-up consultation in the department and did not undergo subsequent POP surgery. Furthermore, we had no information concerning POP-Q points, only POP-Q stage. This can lead to a potential difference in the interpretation of prolapse severity.

A strength of this study is the population size of 1,371 women, which decreases the risk of type 1 error and increases our external validity. In addition, there was a 5-year data registration period, which decreases the risk of unreliable outcome estimates. All women were included based on diagnosis code, action codes, and personal identification numbers, thereby decreasing the risk of selection bias.

## Conclusion

Our study illustrates a high adherence to pessary treatment in women who were found eligible for this treatment by the urogynecologist. However, hysterectomized women and those with incontinence have a significantly lower success rate. Additionally, 38% of women between the ages of 26 and 54 discontinue pessary use owing to personal preference. In the case of prolapse stage and predominant prolapse compartment, no significant difference was found regarding pessary adherence. It appears conclusive to offer a pessary as a first-line treatment in women with symptomatic POP, if the patient agrees to test this treatment and is found eligible by the urogynecologist. Nevertheless, there is still an important need for knowledge as to how best to evaluate and treat women with POP.

## Data Availability

The data that support the findings of this study are available on request from the corresponding author, CHB.
